# Low Advanced Lung Cancer Inflammation Index Predicts Poor Prognosis in Locally Advanced Nasopharyngeal Carcinoma Patients Treated with Definitive Concurrent Chemoradiotherapy

**DOI:** 10.1155/2020/3127275

**Published:** 2020-10-07

**Authors:** Erkan Topkan, Yurday Ozdemir, Ahmet Kucuk, Ozan Cem Guler, Ahmet Sezer, Ali Ayberk Besen, Huseyin Mertsoylu, Sukran Senyurek, Nulifer Kilic Durankus, Yasemin Bolukbasi, Ugur Selek, Berrin Pehlivan

**Affiliations:** ^1^Baskent University Medical Faculty, Department of Radiation Oncology, Adana, Turkey; ^2^Mersin City Hospital, Radiation Oncology Clinics, Mersin, Turkey; ^3^Baskent University Medical Faculty, Department of Medical Oncology, Adana, Turkey; ^4^Koc University, School of Medicine, Department of Radiation Oncology, Istanbul, Turkey; ^5^The University of Texas MD Anderson Cancer Center, Department of Radiation Oncology, Houston, TX, USA; ^6^Bahcesehir University, Department of Radiation Oncology, Istanbul, Turkey

## Abstract

**Purpose:**

We aimed to retrospectively investigate the prognostic worth of pretreatment advanced lung cancer inflammation index (ALI) in locally advanced nasopharyngeal carcinoma (LA-NPC) patients treated with concurrent chemoradiotherapy (C-CRT). *Patients and Methods*. A total of 164 LA-NPC patients treated with cisplatinum-based definitive C-CRT were included in this retrospective cohort analysis. The convenience of ideal pre-C-CRT ALI cut-offs affecting survival results was searched by employing the receiver operating characteristic (ROC) curve analyses. The primary endpoint was the link between the ALI groups and overall survival (OS), while cancer-specific survival (CSS), locoregional progression-free survival [LR(PFS)], distant metastasis-free survival (DMFS), and PFS comprised the secondary endpoints.

**Results:**

The ROC curve analyses distinguished a rounded ALI cut-off score of 24.2 that arranged the patients into two cohorts [ALI ≥ 24.2 (*N* = 94) versus < 24.2 (*N* = 70)] with significantly distinct CSS, OS, DMFS, and PFS outcomes, except for the LRPFS. At a median follow-up time of 79.2 months (range: 6–141), the comparative analyses showed that ALI < 24.2 cohort had significantly shorter median CSS, OS, DMFS, and PFS time than the ALI ≥ 24.2 cohort (*P* < 0.001for each), which retained significance at 5- (*P* < 0.001) and 10-year (*P* < 0.001) time points. In multivariate analyses, ALI < 24.2 was asserted to be an independent predictor of the worse prognosis for each endpoint (*P* < 0.001for each) in addition to the tumor stage (T-stage) (*P* < 0.05 for all endpoints) and nodal stage (N-stage) (*P* < 0.05 for all endpoints).

**Conclusion:**

As a novel prognostic index, the pretreatment ALI < 24.2 appeared to be strongly associated with significantly diminished survival outcomes in LA-NPC patients treated with C-CRT independent of the universally recognized T- and N-stages.

## 1. Introduction

At present, the TNM (tumor-node-metastasis) staging system represents the foremost trustworthy framework for the prognostication of the locally advanced nasopharyngeal carcinoma (LA-NPC) patients undergoing oncologic interventions. However, unfortunately, the comprehensive TNM framework neglects the substantial tumor- and host-related biological differences by relying exclusively upon the locoregional tumor expansions of the primary tumor [1, 2]. These biological differences may instigate divergent clinical outcomes among patients with indistinguishable LA-NPC stages even after the equivalent anticancer interventions, which robustly stresses the specific call for the discovery of novel and more powerful biomarkers for better prognostic categorization of such patients.

Systemic inflammation is appreciated as the seventh hallmark of cancer which provokes cancer initiation, tumor growth, and metastatic dissemination by being an underlying or enabling characteristic that promotes the other six hallmarks [3, 4]. Additionally, the accessible proof has indicated a firm connection between the systemic inflammatory condition and a shortened survival span, as well as many cancer-related symptoms including pain, anorexia, debilitation, and cancer cachexia in numerous cancer types [5]. Therefore, as in other tumor primaries, various investigators focused on the inflammation and nutrition biomarkers in previous LA-NPC studies and identified some factors with notable prognostic utility; these include the body mass index (BMI), C-reactive protein (CRP) and albumin levels, neutrophil to lymphocyte ratio (NLR), platelet to lymphocyte ratio (PLR), Glasgow prognostic score (GPS), and prognostic nutritional index (PNI) [6–11]. Jafri et al. exhibited strong prognostic worth for the advanced lung cancer inflammation index (ALI), as a novel prognostic system, for patients with metastatic non-small-cell lung cancer [12]. The prognostic merit of ALI was also addressed in succeeding esophageal cancer [13], small-cell lung cancer [14], diffuse large B-cell lymphomas [15], pancreatic cancer [16], and head and neck squamous cell cancer [17] investigations and was invariably reported to be an independent prognosticator in these tumor locales too. Interestingly, to date, the prognostic value of ALI has never been addressed in LA-NPC patients who were managed with concurrent chemoradiotherapy (C-CRT). On that account, with this present cohort examination, we expected to explore the prognostic value of ALI in LA-NPC patients who were treated with exclusive C-CRT.

## 2. Patients and Methods

### 2.1. Study Population

We reviewed the institutional medical records of all LA-NPC patients who underwent C-CRT between January 2007 and June 2017 at Baskent University Medical Faculty Department of Radiation Oncology. The following inclusion criteria were established for the investigation: (1) age 18–80 years, (2) Karnofsky Performance Score (KPS) 70–100, (3) histologically documented nonkeratinizing (type 2) or undifferentiated (type 3) squamous cell NPC, (4) clinical/radiological T_3-4_N_0–3_M_0_ or T_1–4_N_1–3_M_0_ disease as per the AJCC 8^th^ ed., (5) BMI ≥ 18.5 kg/m^2^, (6) no history of other cancers, (7) no proof for preceding chemotherapy/RT histories for any reason, (8) receiving at least 1 cycle of platinum-based chemotherapy simultaneously with the RT, (9) accessible pretreatment fluorodeoxyglucose-positron emission computerized tomography (PET-CT), head and neck magnetic resonance imaging (MRI), and chest CT scans, (10) no evidence of brain metastasis in the brain MRI acquired over past 30 days, (11) present evaluable RT and chemotherapy charts, (12) available data of the baseline complete blood count and biochemistry tests, and (13) available records of baseline and follow-up head and neck clinical assessments, MRI and PET-CT scans.

### 2.2. Ethics, Consent, and Permissions

The retrospective study protocol was planned following the specific guidelines outlined in the Declaration of Helsinki and was approved by the institutional review board of Baskent University Medical Faculty before the collection of any patient data. All likely patients gave signed informed consent before the initiation of treatment either themselves or legitimately commissioned representatives for acquisition and analysis of blood samples and pathologic specimens and academic publication of their outcomes.

### 2.3. Concurrent Chemoradiotherapy

Each eligible patient received definitive C-CRT with the RT and chemotherapy doses reported earlier elsewhere [18]. In brief, the RT technique was 3-dimensional conformal RT (3D-CRT) between January 2007 to June 2011 and intensity-modulated RT (IMRT) thereafter, delivered on a daily fractionation basis.

### 2.4. Measurement of Advanced Lung Cancer Inflammation Index

The ALI score was calculated by using the total blood count and biochemistry tests obtained on the first day of C-CRT by using the original formula proposed by Jafri et al. [12]:

(1) $$\begin{array}{c} \text{ALI}=\text{BMI}\times \left(\frac{\text{Albumin}}{\text{NLR}}\right), \end{array}$$

where, BMI is the weight (kg)/height (m)^2^; albumin is the serum albumin in g/dL; and NLR is the neutrophil to lymphocyte ratio.

### 2.5. Response Assessment

We assessed the C-CRT response prospectively even though the study design was retrospective. Each patient was surveyed every 3 and 6 months for the first 2 and 3–5 years and per annum following the C-CRT, or more commonly if clinically necessary. Thorough endoscopic examination was standard for each succeeding visit to ascertain any local/regional relapse(s). The radiological response was evaluated and scored by utilizing the PET-CT scans and the PET Response Criteria in Solid Tumors (PERCIST), respectively. Notwithstanding, when the complete metabolic response was confirmed, the PET-CT scanning was supplanted by the head and neck CT and/or MRI scans, while extra imaging procedures were employed exclusively for the restaging of relapsed disease or suspected lesions. Salvage treatment alternatives or palliative measures including the neck dissection, systemic chemotherapy, radiosurgery, conventional or hypofractionated reirradiation, or their ideal combinations were offered for cases with proven local and/or regional recurrences or distant metastasis (DM), as needed.

### 2.6. Clinical Endpoint

The primary objective of the present retrospective cohort analysis was to assess the probable link between the pre-C-CRT ALI measures and overall survival (OS), defined as the interim between the first day of C-CRT and the date of death or last follow-up. On the other hand, the cancer-specific survival (CSS: the interim between the first day of C-CRT and exclusive NPC-related deaths), locoregional progression-free survival (LRPFS: the interim between the first day of C-CRT and progression or recurrence at the nasopharynx and/or ipsilateral/contralateral neck or death/last follow-up), DM-free survival (DMFS: the interim between the first day of C-CRT and any distant relapses or nonregional lymph nodes or death/last follow-up), and PFS (the interim between the first day of C-CRT and the date of any type of disease progression/death/the last follow-up) as per ALI group comprised the secondary objectives.

### 2.7. Statistical Analyses

The medians and ranges, and frequency distributions were used to describe continuous and categorical variables, respectively. Frequency distributions among the different groups were correlated by utilizing the Chi-square test, Student's *t*-test, or Spearman correlation, as appropriate. The accessibility of pre-C-CRT ALI cut-off that may stratify the study population into two ALI groups with significantly distinct OS and PFS results was evaluated by using the receiver operating characteristic (ROC) curve analysis. Kaplan–Meier estimates and log-rank tests were employed to reveal the likely cooperation between the risk factors and OS and CSS outcomes. In multivariate analyses, the Cox proportional hazards model was utilized to test the independent significance of the factors that exhibited significance in univariate analyses. A 2-sided *P* value < 0.05 was deemed meaningful for intergroup comparisons.

## 3. Results

Present institutional data search identified 209 newly diagnosed LA-NPC patients who underwent C-CRT with at least one chemotherapy cycle between January 2007 and June 2017. However, 164 patients comprised the study population for this current retrospective analysis as 45 cases did not meet the predefined eligibility criteria: 32 undergone induction chemotherapy before the C-CRT, 7 refused the designated chemotherapy protocol, and 6 were lost to follow-up. Pre-C-CRT patient demographics and RT details for the entire study cohort and per ALI score groups are displayed in Table 1. There was no statistically meaningful difference among the groups as the characteristic features were almost evenly distributed between the two ALI cohorts.

**Figure 1:**
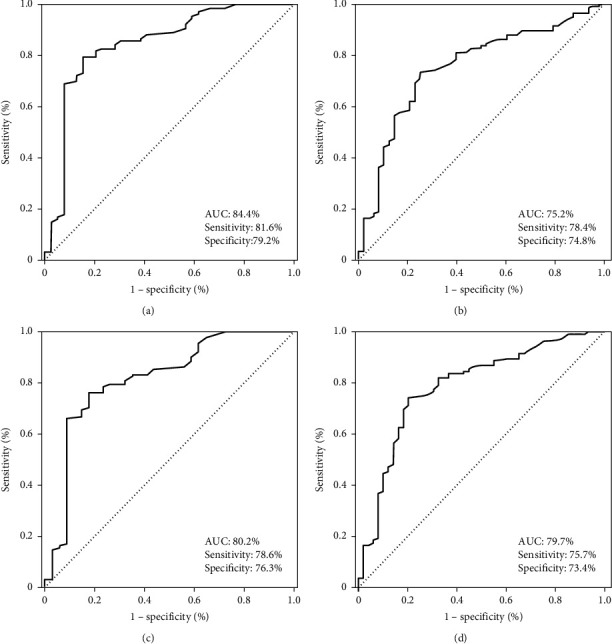
Receiver operating characteristic curve analyses: (a) cancer-specific survival, (b) overall survival, (c) distant metastasis-free survival, and (d) progression-free survival.

Before the survival analysis, first, we sought the convenience of ideal cut-off values which may stratify patients into two significantly separate outcome groups. The median ALI score was 25.4 [95% confidence interval (CI): 22.2–28.6] for the entire research cohort. While no particular discriminatory cut-off value was identifiable for LRPFS outcomes, ROC curve analyses discovered the 24.1 [area under the curve (AUC): 84.4%; sensitivity: 81.6%, specificity: 79.2%], 24.2 (AUC: 75.2%; sensitivity: 78.4%, specificity: 74.8%), 24.0 (AUC: 80.2%; sensitivity: 78.6%, specificity: 76.3%), and 24.2 (AUC: 80.2%; sensitivity: 75.7%, specificity: 73.4%) values as the cut-offs connected significantly with the respective CSS, OS, DMFS, and PFS outcomes (Figure 1). Since all four cut-offs were very close to each other, we fixed the 24.2 value as the ideal ALI cut-off to classify patients into two groups for further analyses: ALI ≥ 24.2 (*N* = 94) versus ALI < 24.2 (*N* = 70), respectively.

At a median follow-up of 79.2 months (95% CI: 62.8–95.6), 116 (70.7%) patients were still alive [ALI < 24.2: 41 (58.6%); ALI ≥ 24.2: 75 (79.8%)], with 105 (65.9%) of them being free of disease progression [ALI < 24.2: 38 (54.8%); ALI ≥ 24.2: 70 (74.5%)]. Locoregional control (LRC) was achieved in 143 (87.2%) cases, while DM was determined to be the leading failure pattern encountered in 39 (23.8%) patients: 25 (35.7%) in ALI < 24.2 and 14 (14.9%) in ALI ≥ 24.2 cohorts (HR: 2.54; *P*=0.001), respectively. As illustrated in Table 2, although there was no difference between the two ALI cohorts concerning the actuarial 5- and 10-year LRC rates, the corresponding actuarial 5- and 10-year DM rates were significantly higher in the ALI < 24.2 group. Overall, 34 of all 48 deaths were reported to be cancer-related, while the remaining 14 deaths were ascribed to causes other than the index NPC, corresponding to a cancer-unrelated death rate of 29.2%.

**Figure 2:**
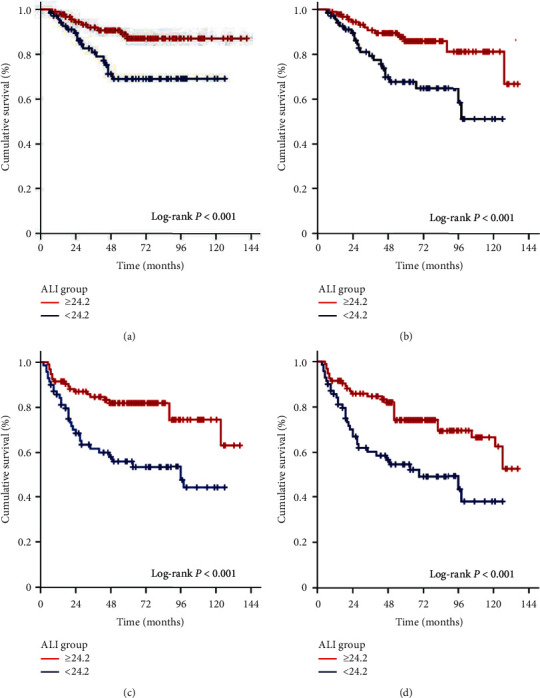
Survival results as per pretreatment advanced lung cancer index (ALI) groups (red line: ALI ≥24.2; dark blue line: ALI <24.2): (a) cancer-specific survival, (b) overall survival, (c) distant metastasis-free survival, and (d) PROGRESSION-free survival.

Median CSS, OS, LRPFS, DMFS, and PFS durations were not reached yet for the whole study accomplice, while the corresponding 10-year survival rates were 79.6%, 68.9%, 66.3, 62.1, and 54.3%, respectively (Table 2). Comparative survival analyses between the ALI cohorts showed that the LA-NPC patients presenting with ALI < 24.2 before the C-CRT course had significantly poorer CSS, OS, DMFS, and PFS than the ALI ≥ 24.2 patients (Figure 2). Corresponding 5- and 10-year survival outcomes were also worse in the ALI < 24.2 patients (Table 2). Presumably being connected with the overall excellent LRC rates mentioned before, LRPFS outcomes appeared to be insignificant between the two ALI cohorts, although the results were numerically favoring the ALI ≥ 24.2 cohort over its ALI < 24.2 counterpart (Table 2).

In univariate analysis, besides the ALI < 24.2 (versus ALI ≥ 24.2; *P* < 0.001), the T3-4 stage (versus T1-2; *P*=0.012) and N2-3 stage (versus N0-1; *P*=0.003) were also identified as the factors to be associated with inferior CSS, OS, DMFS, and PFS results in a statistically meaningful manner (Table 3). An exploratory analysis confined to these prognosticators using a Cox proportional hazards model clearly showed that all three factors were independently influencing the CSS, OS, DMFS, and PFS outcomes (Table 3).

## 4. Discussion

Intensely suggesting a robust independent prognostic merit for pre-C-CRT ALI measures, the outcomes of this retrospective cohort analysis revealed that an ALI value < 24.2 was related to significantly inferior CSS, OS, DMFS, and PFS results in LA-NPC undergoing conclusive C-CRT. Therefore, as it conjointly reflects the immune, nutritional, and inflammatory status of the affected patient, the cost-effective and easy to calculate ALI may be useful for more accurate prognostic lamination of the LA-NPC patients, which may further guide the proper selection of the fittest treatment choices for such patients when used in collaboration with the traditional TNM staging framework.

Mounting evidence has proven that the systemic inflammation, the seventh hallmark of cancer, plays critical roles all through the initiation to metastasis steps of the carcinogenesis process [19]. Other than being an initiator of carcinogenesis in >25% of all solid or hematologic cancers [20, 21], chronic inflammation additionally provides a favorable state for increased proliferation and survival of tumor cells by inciting the neoangiogenesis, antiapoptotic, and immune escape pathways, as well as by expediting the metastasis to regional and remote sites, and induction of a highly resistant tumor phenotype against anticancer therapies, including the RT and chemotherapy [19, 22, 23]. Given such conclusive fundamental proof, multiple blood-borne biomarkers of the systemic inflammatory response, either each one seperately or in various blend forms, have been searched for their prognostic usefulness in patients with various cancers and disease stages, with published results harmoniously complimenting a solid connection between these markers and patients' prognoses irrespective of the disease stage [24]. As lately stated by Jafri et al., ALI represents one such unique blend that consolidates the BMI, albumin, and NLR, which demonstrated significant value in the prognostic lamination of patients presenting with various cancers [12–17, 25]. Nevertheless, regardless of the convenience of dependable proof for the chronic inflammation either as a carcinogenesis initiator and/or as an accelerator of disease progression for NPCs, prompting the planning of our current study, ALI has never been studied before in LA-NPC patients managed with C-CRT for its prognostic merit.

The most noticeable finding of our present study was the first time successful exhibition of a vital incentive for the pre-C-CRT ALI in prognostic stratification of LA-NPC patients into two distinct groups treating the CSS, OS, PFS, and DMFS, but not LRPFS, results. Although it is difficult to dependably remark on the genuine value of these exceptional discoveries in absence of similarly designed ALI research, they seem to concur well with the published results of ALI studies for other tumor sites [12–17, 25] and the NPC studies examining the prognostic utility of BMI, albumin, and NLR [6–8], namely, the components of ALI. Previously, Huang et al. investigated the link between the pretreatment BMI and the clinical outcomes in patients with LA-NPC treated with C-CRT in a cohort of 400 patients [6]. The researchers reported that the 5-year PFS rates for the underweight, normal weight, overweight, and obese groups were 44%, 61%, 68%, and 73%, respectively (*P*=0.014), and the 5-year OS rates were 51%, 68%, 80%, and 82% (*P*=0.001), individually. Because the BMI was shown to be an independent prognosticator on both survival endpoints, the authors inferred that the BMI was a simple and steady independent prognostic factor for patients with LA-NPC undergoing definitive C-CRT. Likewise, in a more recent propensity score-matched report, Ouyang et al. investigated the impact of pre-C-CRT BMI on outcomes of 1778 LA-NPC patients treated with the IMRT technique [26]. Stratification of patients into underweight (<18.5 kg/m^2^), normal weight (18.5–22.9 kg/m^2^), overweight (22.9–27.5 kg/m^2^), and obese (≥27.5 kg/m^2^) as per their BMI measures indicated that the low BMI was altogether linked with significantly poorer CSS/OS (*P*=0.042) and DMFS (*P*=0.025) results independent of the other customary TNM-related factors. In a very recent meta-analysis, Yang et al. assessed the prognostic significance of pretreatment serum albumin in 10 NPC studies comprising 7339 patients [7]. Implying an excellent prognostic merit for pretreatment albumin measures, the results of this comprehensive meta-analysis showed that lower serum albumin levels were connected with significantly worse OS (HR = 1.32; *P* < 0.001) and DMFS (HR = 1.40; *P* < 0.001) results. Considering the NLR, Yin et al. [8] conducted a large meta-analysis including a total of 6 studies and 4359 LA-NPC patients. The authors stated that an elevated pretreatment NLR was associated with poorer OS (HR = 1.74; *P* < 0.01) and PFS (HR = 1.48; *P* < 0.01) in their pooled cohort analyses, which besides seemed, by all accounts, to be uninfluenced by the utilization of various NLR cut-offs (<3 or ≥3). In short, the results of these large-scale studies and comprehensive meta-analyses displayed noteworthy prognostic utility of each component of the ALI in particular manners. Compared to these, our current outcomes appeared to wisely propose that the ALI had the potential to be a more robust prognostic index than its separate constituents, as our *P* values for each of the CSS, OS, DMFS, and PFS (*P* < 0.001for each) were superior to the ones accomplished in the aforementioned studies. In spite of the necessity for affirmation of our current outcomes, this superiority might be a reflection of the ALI's unique power in provision of the host's systemic inflammation, immune, and nutritional status in a simultaneous manner compared to each of its separate constituents.

Compared to the most assessable LA-NPC studies scarcely addressing the noteworthiness of various prognostic indices in LA-NPC patients, we additionally analyzed the relation between the ALI and the CSS and LRPFS. Our critical research indicated a significant connection between a low ALI and notably inferior CSS, but not LRPS, outcomes. Reckoning that almost 40% of all mortalities are assigned to the intercurrent chronic illnesses or different causes as opposed to the LA-NPC itself [27], which was 29.2% in our present research, such kind of research may have utmost significance in the dismissal of perplexing cancer-unrelated causes and thorough evaluation of the definite merit of the intended prognostic factors or indices in this patients' group. Moreover, 90% higher locoregional control rates revealed from the IMRT literature for LA-NPC and our overall 10-year actuarial rate of 87.6% [11, 28, 29] altogether do not only disclose the most likely reason for the lack of a meaningful relationship between the ALI measures and LRPFS, but also robustly stress the pressing urgency for the implementation of more effective systemic agents to the current LA-NPC treatment algorithms to accomplish superior survival outcomes, such as the novel targeted agents and/or immunotherapies.

The precise mechanisms underneath the multifaceted relationship between a low ALI score and inferior survival outcomes have not been explained to date. Still, some hypothetical but sensible remarks can be made by evaluating the connection between each key component of the ALI and the survival outcomes of NPC patients. To begin with, BMI is calculated by utilizing the patient's constant height and varying weight measures. Either weight loss (particularly >5% at the past 6 months) or allied lessened BMI has been separately shown to be firmly linked with precachectic/cachectic body composition [30, 31], which has been established to indicate increased mortality rates in all cancer types, including the LA-NPC [32–34]. Similarly, lower albumin levels symbolize insufficient nutritional and/or hypercatabolic states which result in weight loss and lowered BMI in cancer patients [35, 36]. Low albumin levels additionally reflect an aggravated inflammatory status, as it is almost invariably associated with increased measures of C-reactive protein [37, 38]. Moreover, low albumin levels can impair the antioxidant actions against carcinogens, cellular and humoral immunity, and cellular phagocytic functions; stimulate the DNA replication in cancer cells; and resultantly enhance tumor growth rates [35–38]. Regarding the NLR, it is well proven that elevated numbers of circulating neutrophils are associated with poor patients' prognoses as a result of the secretion of the proangiogenic and antiapoptotic chemokines, which may facilitate the tumor growth and metastatic potential [8]. Strikingly contrasting with the neutrophils, lymphocytes represent the immune cells with antitumor functions [8]. Thus, an elevated NLR indicates increased neutrophil and/or decreased lymphocyte counts and, therefore, hyperinflammatory and suppressed immune status, which is undoubtedly associated with poor prognosis in cancer patients. Notified with these fundamental and clinical data, the prognostic power of ALI appears to represent a composite result of its ingredients' unique prognostic power. Besides, as we have initially homogenized our cohort by excluding underweight patients (BMI <18.5 kg/m^2^), our data might be also noted as “corrected ALI for BMI” to reveal the outcome directly related to the inflammatory and immunologic state in otherwise fit group of patients.

The present study is strengthened by some key factors: homogenous staging procedure with PET-CT and MRI, standard treatment with exclusive C-CRT comprising identical chemotherapy and RT regimes, and measurement of all components of the ALI, necessarily carried out at the same time point (first day of C-CRT), namely, the BMI, albumin, and absolute neutrophil and lymphocyte counts. However, our present study also has some certain drawbacks. First, it is a single institutional cohort analysis in a comparably small study population where the information was based on retrospective chart reviews; in consequence, several unpredictable tumor- or patient-related factors may have inadvertently altered the results in favor of one group. Second, all ALI scores presented here were just the reflections of the measurements and resulting calculations at a single time point: the first day of the C-CRT. However, ALI is a dynamic biological marker that exhibits marked fluctuations at any time point during the C-CRT and posttreatment follow-up periods depending on the impending timely changes in the overall tumor load including the measurable locoregional tumor primary and indemonstrable but conceivably present microscopic metastatic tumor foci, the affected hosts' systemic inflammation response, and immunity status. Therefore, ensuing investigations focusing on the time-dependent changes of ALI, so-called “ALI dynamics,” might prove useful by facilitating the identification of more relevant cut-off(s), such as the “ALI nadir” or “ALI peak,” and potentially more reliable prognostic lamination of the LA-NPC patients undergoing exclusive C-CRT. Third, the lack of a thorough evaluation of the additional immune, inflammation, and nutritional chemokines and cytokines, like the C-reactive protein, IL-6, anemia status, and weight loss at the past 6 or 12 months restricted our ability to resolve the potentially valuable interactions between the ALI and these markers. Therefore, the results of this first effort investigating the prognostic significance of ALI in LA-NPC patients treated with C-CRT should be granted as hypothetical and ought to be asserted with suitably designed further research for more solid conclusion on the true prognostic merit of ALI in such patients.

## 5. Conclusion

The discoveries of this first endeavor retrospective cohort analysis evaluating the prognostic utility of ALI in LA-NPC patients undergoing C-CRT recommended that a pre-C-CRT ALI value <24.2 was independently equated with poorer CSS, OS, DMFS, and PFS outcomes in such patients' groups.

## Figures and Tables

**Table 1:** Baseline and treatment characteristics of all study population as per advanced lung cancer inflammation index group.

Characteristics	All patients (*N* = 164)	ALI ≥24.2 (*N* = 94)	ALI <24.2 (*N* = 70)	*P* value
Median age (years)	56	57	54	0.59
Range	27–79	27–79	31–76	—
Age group (*N*; %)				
≥70 years	37 (22.6)	20 (21.3)	17 (24.3)	0.67
<70 years	127 (77.4)	74 (78.7)	53 (75.7)	
Gender (*N*; %)				
Male	129 (78.7)	73 (77.8)	56 (80.0)	0.79
Female	35 (21.3)	21 (22.2)	14 (20.0)	
KPS (*N*; %)				
90–100	72 (43.9)	44 (46.8)	2840.0)	0.35
70–80	92 (56.1)	50 (53.2)	42 (60.0)	
WHO histology (*N*; %)				
2	19 (11.6)	11 (11.7)	8 (13.4)	0.92
3	145 (88.4)	83 (88.3)	62 (88.6)	
T-stage (*N*; %)				
1-2	25 (15.2)	15 (16.0)	10 (14.3)	0. 76
3-4	139 (84.8)	79 (84.0)	60 (85.7)	
N-stage (*N*; %)				
0-1	46 (28.0)	28 (29.8)	18 (25.7)	0.53
2-3	118 (72.0)	66 (70.2)	52 (74.3)	
Clinical stage (*N*; %)				
II	19 (11.6)	11 (11.7)	8 (12.5)	0.63
III	81 (49.4)	48 (51.1)	33 (47.1)	
IVA-B	64 (39.0)	35 (37.2)	29 (41.4)	
RT technique				
3D-CRT	52 (31.7)	29 (30.9)	23 (32.9)	—
IMRT	112 (68.3)	65 (69.1)	47 (67.1)	

ALI: advanced lung cancer inflammation index; KPS: Karnofsky Performance Score; WHO: World Health Organization; T-stage: tumor stage; N-stage: nodal stage.

**Table 2:** Survival and tumor control results as per advanced lung cancer inflammation index group.

Outcome	All patients (*N* = 164)	ALI ≥24.2 (*N* = 94)	ALI <24.2 (*N* = 70)	*P* value
CSS				
Median (mo)	NR	NR	NR	<0.001
5-year (%)	79.6	87.1	69.0	
10-year (%)	79.6	87.1	69.0	
OS				
Median (mo)	NR	NR	NR	<0.001
5-year (%)	74.4	84.6	67.7	
10-year (%)	68.9	80.9	52.1	
LRPFS				
Median (mo)	NR	NR	NR	0.17
5-year (%)	72.6	83.4	62.1	
10-year (%)	66.3	75.3	55.8	
DMFS				
Median (mo)	NR	NR	69.6	<0.001
5-year (%)	71.1	81.6	57.8	
10-year (%)	62.1	76.3	47.6	
PFS				
Median (mo)	NR	NR	63.2	<0.001
5-year (%)	66.5	75.7	54.7	
10-year (%)	54.3	65.3	38.6	
Actuarial LRC				
5-year (%)	146 (89.0)	88 (93.6)	58 (82.6)	0.18
10-year (%)	143 (87.2)	87 (92.6)	56 (80.0)	0.15
Actuarial DM				
5-year (%)	37 (22.6)	14 (14.9)	23 (32.9)	<0.001
10-year (%)	39 (23.8)	14 (14.9)	25 (35.7)	<0.001

ALI: advanced lung cancer inflammation index; CSS: cancer-specific survival; OS: overall survival; DMFS: distant metastasis-free survival; PFS: progression-free survival; LRC: locoregional control; DM: distant metastasis.

**Table 3:** Results of uni- and multivariate analysis.

Factor	CSS			OS			DMFS			PFS		
	Univariate *P* value	Multivariate *P* value	HR	Univariate *P* value	Multivariate *P* value	HR	Univariate *P* value	Multivariate *P* value	HR	Univariate *P* value	Multivariate *P* value	HR
Gender (M vs. F)	0.64	—	—	0.76	—	—	0.79	—	—	0.61	—	—
Age group (<70 vs. ≥ 70 y)	0.41	—	—	0.37	—	—	0.53	—	—	0.64	—	—
KPS (90–−100 vs. 70–80)	0.47	—	—	0.53	—	—	0.39	—	—	0.55	—	—
Histology (2 vs. 3)	0.87	—	—	0.76	—	—	0.82	—	—	0.84	—	—
T-stage (1-2 vs. 3-4)	0.012	0.08	1.23	0.021	0.009	1.19	0.008	0.006	1.21	0.018	0.08	1.19
N-stage (0-1 vs. 2-3)	0.003	0.006	1.48	0.005	0.007	1.38	0.002	0.001	1.62	0.005	0.002	1.40
ALI (≥vs. <24.2)	<0.001	<0.001	1.93	<0.001	<0.001	2.32	<0.001	<0.001	3.87	<0.001	<0.001	3.21

CSS: cancer-specific survival; OS: overall survival; DMFS: distant metastasis-free survival; PFS: progression-free survival; M: male; F: female; KPS: Karnofsky Performance Score; T: tumor; N: node; ALI: advanced lung cancer inflammation index.

## Data Availability

The datasets used and/or analyzed during the current study are available from the Baskent University Department of Radiation Oncology Institutional Data Access for researchers who meet the criteria for access to confidential data, through the following contact address: adanabaskent@baskent.edu.tr.
